# Terrell Medical Society

**Published:** 1894-02

**Authors:** 


### Society Notes.

### TERRELL MEDICAL SOCIETY.

This organization, we are pleased to say, is in a prosperous condition, the members taking a lively interest in its meetings. At a recent meeting, well attended, the following officers were elected:

President, Dr. B. F. Church, First Assistant Physician State Lunatic Asylum, elected by acclamation.

First Vice-President, Dr. J. A. Anthony.

Second Vice-President, Dr. J. M. Payne.

Secretary, Dr. W. I. Swangen.

Dr. L. M. Stroud read a very able paper, having for its subject typhoid fever. The paper was quite freely discussed, and each member gave his form of treatment for this disease. Dr. Preston suggested that Dr. Stroud's paper be published.

The remainder of the program was dispensed with until next meeting.

Dr. B. F. Powell, the retiring President, made a brief and appropriate farewell address, and then conducted his successor to the chair, who in a few happy words thanked the society for the honor conferred.

The President appointed the following committees: New Remedies and Diseases, Drs. Anthony, Preston and Dumas; Committee on Publication, Drs. Monday, Stroud and Preston. Preparatory to adjournment for the banquet, the President appointed Dr. J. A. Anthony as master of ceremonies for the occasion.

### THE BANQUET.

At 2 o'clock the Society repaired in a body to the dining parlor of the Harris House, where the twelfth semi-annual banquet of the Terrell Medical Society was held. The capacious hall was filled with a dozen tables, burdened with the weight of viands, luscious fruits, etc., around which were seated the members of the Society, with their ladies and quite a number of visitors. At the conclusion of the sumptuous feast, the master of ceremonies arose and called upon Dr. Manson, of Rockwall, for a toast. That gentleman very adroitly shifted this duty to Dr. Preston, who retaliated as follows:

"This is an occasion where wit should prevail, and as Dr. Manson is possessed of all of it, he is the proper person to lead in the matter."

Drs. Dumas, Monday, Church and Anthony made short and felicitous speeches. At this juncture, Dr. P. M. Payne was called upon, who gathered in both hands a large and beautiful bouquet, which he presented to Dr. B. F. Church, in a very happy way. The bouquet was a gift from Mrs. Preston. Dr. Church responded gracefully to the presentation speech.

Dr. Manson was again called on, and favored the Society with one of his entertaining talks, at the conclusion of which the Society and its guests repaired to the parlors of the hotel, where a short time was pleasantly spent in conversation.

Those in attendance on the banquet were: Dr. B. F. Church and Miss Ethel Cowles, Dr. Laurie MacKechney and Miss Kate Childress, Dr. P. M. Payne and Miss Lucile Grinnan, Dr. G. F. Powell and lady, Dr. J. A. anthony and lady, Dr. W. S. Sanders and lady, Dr. W. P. Dumas and lady, Dr. W. H. Monday, Miss Jewel Monday, Miss Nannie Powell, Miss Zula Byrn, Drs. Preston, Manson, Roberts, Penn, C. M. Crumbaugh, and Charles and Albert Monday.

